# Low pressure headache and cerebral fat embolism from a sacral fracture through a Tarlov cyst: a case report

**DOI:** 10.1186/s13256-023-04142-2

**Published:** 2023-10-07

**Authors:** Delran Anandkumar, Umayr Jakhura, Kathleen Potter, Ibrahim Bhatti

**Affiliations:** NHS England, London, UK

**Keywords:** Tarlov Cyst—fluid filled nerve root cysts, Embolism—a material causing a blockage inside of a blood vessel, Neoplasm—Abnormal growth of tissue, Meningitis—Infection of lining around the spine and brain, Trigeminal autonomic cephalalgias—headache disorders that are caused by unilateral trigeminal distribution pain, Photophobia—increased sensitivity to light, Analgesia—medications which relieve pain, Insufficiency fractures—type of stress fracture

## Abstract

**Background:**

Here we report the only formally documented case in the United Kingdom, to our knowledge, of a cerebral fat embolism secondary to non-iatrogenic trauma through a Tarlov cyst. This case demonstrates the pathology clearly giving an excellent opportunity to demonstrate a rarely seen pathology as well as illustrating the importance of the patient history to guiding further management.

**Case presentation:**

A middle-aged patient was admitted on the acute medical take complaining of severe headache with photophobia, having just returned after a skiing holiday. Computerised tomography scan of the head showed fat within the anterior horn of both lateral ventricles, and within the subarachnoid space. Re-discussion with the patient and subsequent MRI (Magnetic Resonance Imaging) of the spine identified the pathogenesis of her symptoms: a sacral insufficiency fracture through a Tarlov cyst, causing subarachnoid fat embolism and symptoms of a low-pressure headaches due to a dural leak. Patient was medically managed and discharged with planned follow-up. Due to the Coronavirus pandemic and resolution of the patient's symptoms, they declined further follow up imaging.

**Conclusions:**

The case demonstrates a rarely seen pathology as cause of a common presenting problem, headache. Emphasizing the importance of history taking and appropriate investigations in medical cases that do not conform to the usual diagnosis.

## Background

Headaches are a common presenting complaint in acute medical units across the country. Whilst this case report demonstrates is a rare constellation of symptoms, the prevalence of Tarlov cysts in one study was 4.6% in patients with back pain [1]. This can be an underlying cause of spinal headaches/focal neurology in patients presenting to the acute medical unit and must be considered. Albeit many patients coming in with convincing red flags will have a CT head, it is important to consider imaging of the axial skeleton if there are signs of subarachnoid fat embolism on CT.

## Case presentation

A 65-year-old retired Chinese female patient presented to the emergency assessment unit with a 3-day history of a band like headache, 9/10 in severity. The pain was worse around the eyes, exacerbated by standing but improved on lying down. Light made the pain worse. However, there were no other features of meningism and no focal neurology noted in the history.

Her past medical history included a left frontal cavernoma, high cholesterol, fibroids and fatty liver. She worked as a part time languages teacher.

On examination observations were within normal parameters.

### Examinations

Glasgow Coma Scale: E4V5M6.

Cranial nerve examination: no focal neurology. Pupils equal and reactive.

Peripheral nervous system examination: normal (Table 1).

**Table 1:** Peripheral nervous system examination

	Upper limbs		Lower limbs	
	Right	Left	Right	Left
Tone	Normal	Normal	Normal	Normal
Power	5/5	5/5	5/5	5/5
Co-ordination	Normal	Normal	Normal	Normal
Reflexes	+	+	+	+

Down-going planters’.

Digital rectal examination: normal sensation.

Heart: no acute findings.

Lungs: no acute findings.

Abdomen: no acute findings.

## Investigations

**Fig. 1:**
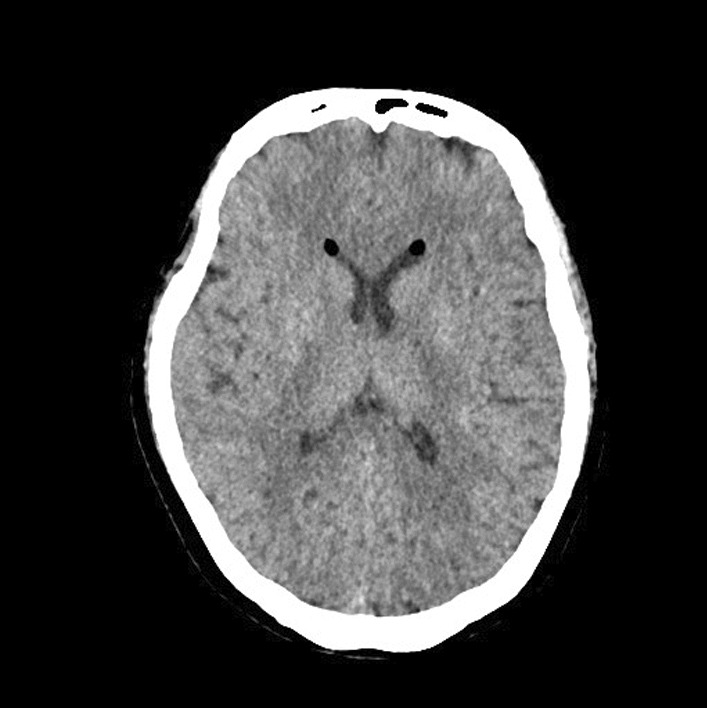
Computerised tomography scan showing incidental fat within the anterior horn of both lateral ventricles

A CT scan was arranged in view of the history and symptoms. This showed the following (Fig. 1):

fat within the anterior horn of both lateral ventricles, and within the subarachnoid space in posterior fossa and around the circle of Willis.

This unusual finding on CT resulted in the team re-taking the history with the patient. On further questioning of the timescale of the headaches, the patient explained she had returned from a skiing holiday 3 days ago. She described a particular event after which the headaches had started. She had been skiing when she slipped, falling onto her bottom (no head injury). At the time of the fall, she did not injure herself and was able to get up and mobilize with no issues. That evening she started having headaches which resumed the following day in the airport following her flight back to the UK.

On re-examination of the patient:

*Cervical-spine*: no tenderness or reduced range of movement

*Spine*: Tenderness at sacral level of spine on deep palpation. Normal range of movement of spine. No paraspinal tenderness.

**Fig. 2:**
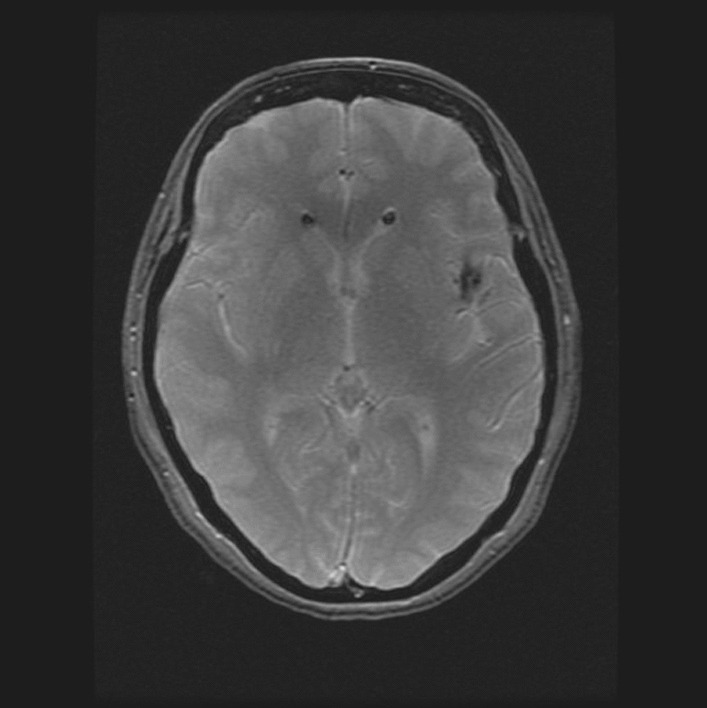
Gradient echo sequence showing intraventricular fat and incidental left frontal cavernoma

**Fig. 3:**
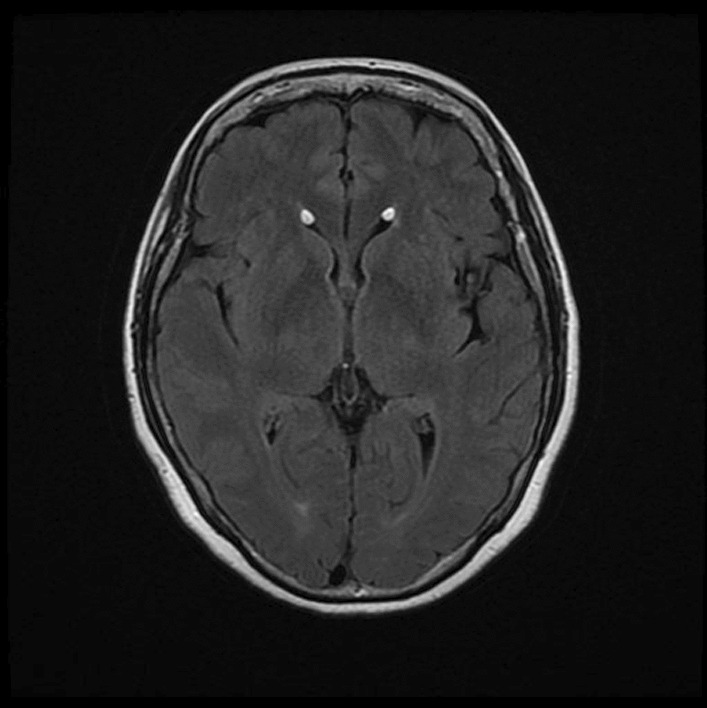
T2 flair image showing fat embolism

**Fig. 4:**
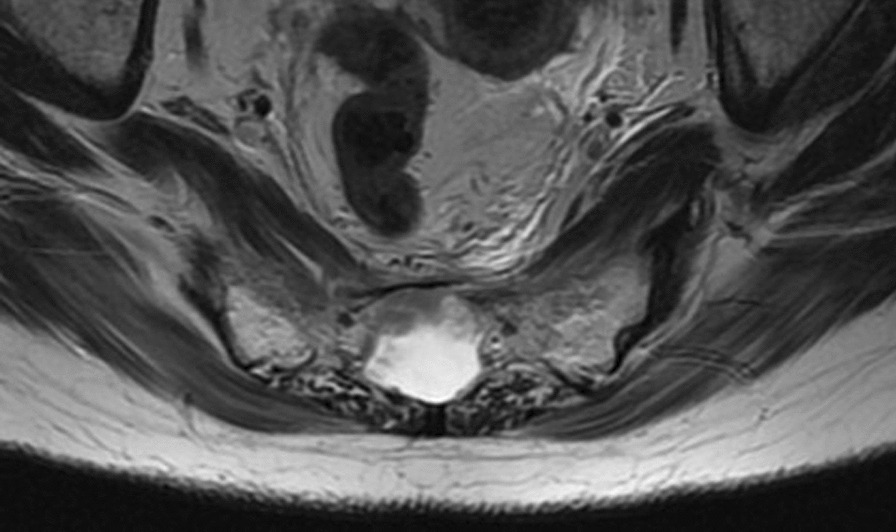
Axial T2 sacral fracture

**Fig. 5:**
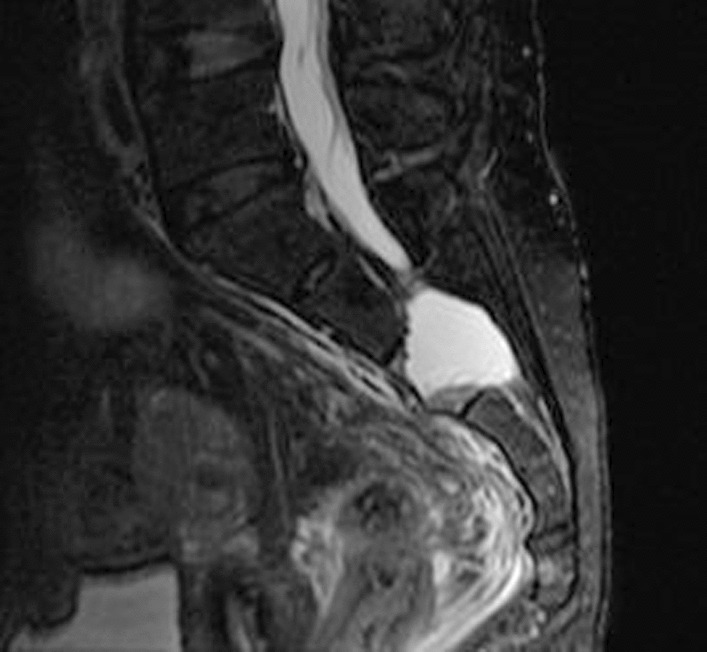
Sagittal STIR sacral fracture

Subsequently, having discussed examination findings with the radiology team, we requested MRI head and MRI spine (Figs. 2, 3, 4 and 5). The findings were as follows:

MRI head: “*Resolving intraventricular and subarachnoid fat. The relative speed of resolution suggests a precipitating incident. Is there any evidence of recent vertebral fracture?*”

MRI spine: “*Sacral fracture through Tarlov cyst at S3. This is presumed to be the source of the intra cerebral fat embolism.*”

## Differential diagnosis

British Medical Journal Best Practise released guidance on the assessment of acute headaches in adults. In this document, 11 differentials were listed as common whilst 23 were listed as uncommon [2]. Of these vast range of differentials, our cause of headache was not listed illustrating how rare it is in these circumstances. The pertinent factors in the history included photophobia, headache worsened by standing up and a previously noted cavernoma.

Of these, it would be prudent to exclude: [2, 3]

•Vascular: subarachnoid haemorrhage/intracranial haemorrhages including bleed relating to the known cavernoma/central venous thrombosis.
•Non-vascular intracranial disorders: neoplasm.
•Infection: Meningitis.
•Other: migraines/cluster headaches/trigeminal autonomic cephalalgias.

Due to the cavernoma and photophobia, the CT head was useful to rule out vascular cause for headache, in particular any intracerebral bleeding. Infective causes were less likely as the patient did not display any clinical or biochemical markers of infection. The history also was felt too short lived to suggest underlying neoplasm.

The unusual CT head findings prompted further history taking, in particular focusing around events leading up to the start of the headache. Also, in discussion with our Radiology colleagues, more targeted and focussed investigations were requested, namely MRI of the sacral spine.

## Treatment

Patient medically managed. Regular analgesia for headache and encouraged to maintain good hydration No further treatment required and follow up arranged to monitor the patient’s symptoms.

## Outcome and follow-up

In our case, the patient felt well, with the headache beginning to resolve prior to MRI head and sacrum. She was discharged home with referral to the specialist orthopaedic pelvic team for follow up and repeat CT scan in 6 weeks. However, due to the Coronavirus pandemic, the scan was declined by the patient. We called the patient 4 months later to find they were completely asymptomatic with no recurring symptoms since discharge.

## Discussion and conclusions

Headaches represent a challenge to acute medical units up and down the country, causing anxiety for both patient and doctor out of fear of missing something serious as a cause. More often than not these headaches will have a benign origin and be due to a common differential: “Primary headache disorders—migraine, tension headache and cluster headache—constitute nearly 98% of all headaches” [4].

However in this instance, an unusual pathology was the cause. Valuable information was elicited from the a thorough history and analysing specific symptoms the patient mentioned, enabling reasonable differentials to be produced.

In this case, a number of factors came together to produce a pathological process which is not common.

The main factor that made this presentation possible was the fact this patient had a Tarlov cyst in situ. Tarlov cysts (perineural cysts) were first described in 1938 by Tarlov during autopsy’s. They were initially described as being located in the posterior sacral/coccygeal nerve roots, most often the second or third, (and rarely the thoracic spine). Tarlov cysts are filled with cerebrospinal fluid and generally stable in size. There is no clear pathogenesis of these cysts [1].

Clinical symptoms from Tarlov cysts range from being completely asymptomatic to localized pain, neurological disturbances such as weakness or bowel and bladder incontinence (due to pressure effects) and insufficiency fractures [1], as occurred in this particular patient.

The relatively minor fall the patient suffered resulted in an insufficiency fracture through the Tarlov cyst at S3, causing a low pressure headache. Tarlov cysts can have nerve fibres running through the cyst walls therefore complications include neurological deficits, urinary disturbances, infection and spinal headaches [1]. Specifically in this case, the disruption of the cyst (due to the fracture) caused spinal headaches as a result of a dural leak. This caused the specific symptom of pain worse on standing and better on lying down.

The final phenomena identified in this case was the fat embolism. Normally fat embolus is associated with long bone fractures to lower extremities with subsequent embolism to the brain (and occasionally fat embolism to the middle cerebral artery during mitral valve replacements) [5]. Fat embolism typically results in three particular symptoms: petechial rash, respiratory symptoms and/or central nervous system signs [6].

Another type of fat embolism, which is more relevant in this particular case, is SFE (Subarachnoid Fat Embolism) which affects the central nervous system primarily. Issues as a result of SFE can include hydrocephalus, aseptic meningitis, meningeal calcifications and seizures—these resulting conditions can be transient and return to baseline [6]. The presence of fat attenuation droplets in the subarachnoid space is rarely discussed in literature, however, intraspinal dermoids/teratomas have been implicated as a source of fat emboli [5]. On the scans itself, the fat will be situated in the non-dependent sites as fat is less dense than cerebrospinal fluid [5]. SFE can occur spontaneously/after surgical management of neuroaxis. It is however less commonly reported after trauma [6].

Fortunately in the case of our patient, there were no complications directly from the subarachnoid fat embolism, but rather from the sacral fracture and the subsequent dural leak. The causative agent in this case was trauma resulting in the insufficiency fracture through the Tarlov cyst and subsequent dural leak.

In a study involving 500 patients with lower back pain who had an MRI scan, there was a prevalence of 4.6% of patients (23 in total) with underlying Tarlov cysts [1]. For patients with symptomatic Tarlov cysts, the management of choice is simple analgesia and physiotherapy. If this were to fail, surgical management is the next stage with various techniques being employed. There is “no consensus on the appropriate surgical indications and techniques but percutaneous drainage or microsurgical excision combined with duraplasty or plication of the cyst wall appear to be effective and safe” [7].

## Take Home Messages

- Thorough history taking to guide investigations
- The importance of inter disciplinary discussion with colleagues of challenging cases especially with findings outwith the norm. Guidance from our Radiology colleagues was essential to help us understand the cause of the patient's headache in this case and confirm the diagnosis
- Tarlov cysts can be an underlying cause of lower back pain and can cause complications if trauma occurs
- Important to consider imaging of the axial skeleton if there are signs of subarachnoid fat embolism on CT head for patients presenting with a fall.

## Data Availability

All data generated or analysed during this study are included in this published article with referencing.

## Abbreviations

CT Scan: Computerised tomography scan
MRI Scan: Magnetic resonance imaging scan
SFE: Subarachnoid fat embolism
